# Exceptional Location of a Papillary Carcinoma of the Thyroid

**DOI:** 10.7759/cureus.43213

**Published:** 2023-08-09

**Authors:** Afaf Thouil, Meriem Rhazari, Amal Bennani, Hatim Kouismi

**Affiliations:** 1 Department of Respiratory Diseases, Research and Medical Sciences Laboratory, Faculty of Medicine and Pharmacy of Oujda, Mohammed VI University Hospital, Mohammed First University, Oujda, MAR; 2 Department of Pulmonology, Faculty of Medicine and Pharmacy of Oujda, Mohammed VI University Hospital, Mohammed First University, Oujda, MAR; 3 Department of Anatomopathology, Faculty of Medicine and Pharmacy of Oujda, Mohammed VI University Hospital, Mohammed First University, Oujda, MAR; 4 Department of Pneumology, Faculty of Medicine and Pharmacy of Oujda, Mohammed VI University Hospital, Mohammed First University, Oujda, MAR

**Keywords:** surgery, trachea, larynx, carcinoma, thyroid

## Abstract

Thyroid cancers are a rare condition; of these, differentiated thyroid carcinomas are the most common and have a good prognosis with timely diagnosis and treatment. In the case of a late diagnosis, these carcinomas can breach the thyroid capsule and invade the laryngo-tracheal axis. The surgical treatment of locally invasive papillary thyroid carcinoma is a complete resection with the preservation of laryngeal functions when it is possible. We report the case of an 80-year-old patient who presented with inspiratory dyspnea and hemoptysis showing endotracheal localization of a papillary carcinoma of the thyroid.

## Introduction

Papillary carcinoma of the thyroid is the most common type of thyroid cancer, usually characterized by slow growth and excellent long-term survival [1]. However, this cancer can sometimes present in unusual ways, defying clinical expectations and requiring tailored management. In this case report, we present an exceptional localization of a papillary carcinoma of the thyroid, illustrating the diversity of clinical presentations of this disease. Our aim is to highlight this atypical presentation, examine the diagnostic and therapeutic challenges it poses, and discuss the implications for the clinical management of patients.

## Case presentation

An 80-year-old male patient, a chronic smoker with a history of 30 pack-years, now abstinent, with ischemic heart disease under treatment, presented with moderate hemoptysis and inspiratory dyspnea evolving over two months in a context of general health deterioration.

The clinical examination results showed a patient with an ECOG performance status of 3, correctly saturating in ambient air with wheezing on auscultation without any other associated respiratory or extra-respiratory signs. A cervical examination revealed the presence of right lateral cervical lymph nodes, however, it did not reveal any mass or induration in the thyroid area. 

**Figure 1:**
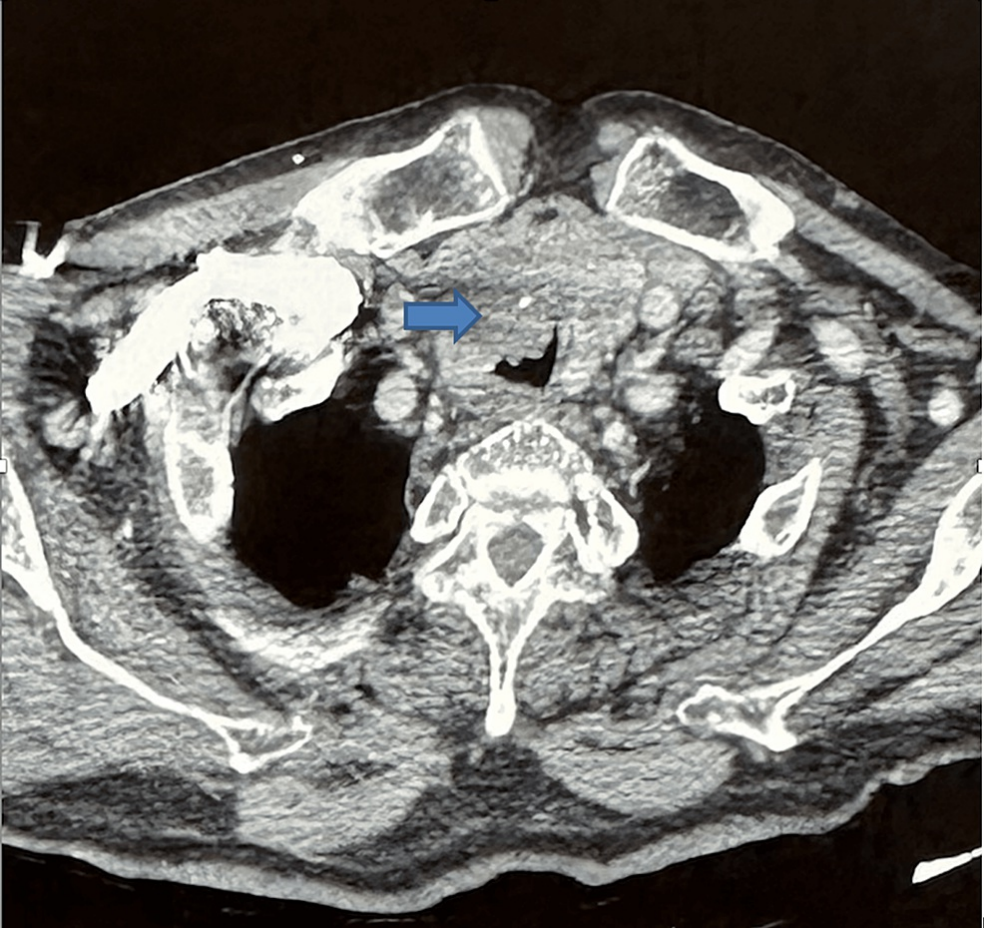
The CT scan revealed heterodense tissue lesion budding into the trachea at the level of D2, reducing the tracheal lumen and seeming to continue with the right isthmo-lobar thyroid parenchyma(blue arrow).

The cervical and thoracic computed tomography scan (Figure 1) revealed an irregular, heterodense tissue lesion budding into the trachea at the level of D2, reducing the tracheal lumen and continuing into the right isthmo-lobar parenchyma, which is heterogeneous and contains calcifications. Necrotic lateral cervical lymph nodes are present, some measuring 29x10mm and 20x16mm at the level of the left IV chain. Bilateral parenchymal lung nodules and micronodules are also present.

Thyroid function tests were within the euthyroid range. As part of the extension assessment, a nodular lesion of the occipital horn of the right lateral ventricle, with regular contours, well-defined, non-enhanced was found.

Brain MRI shows an intraventricular nodular formation at the level of the occipital horn of the right lateral ventricle, containing a tissue component in hypo signal T1 enhanced after gadolinium injection. This could be related to a secondary lesion, given the patient's context.

**Figure 2:**
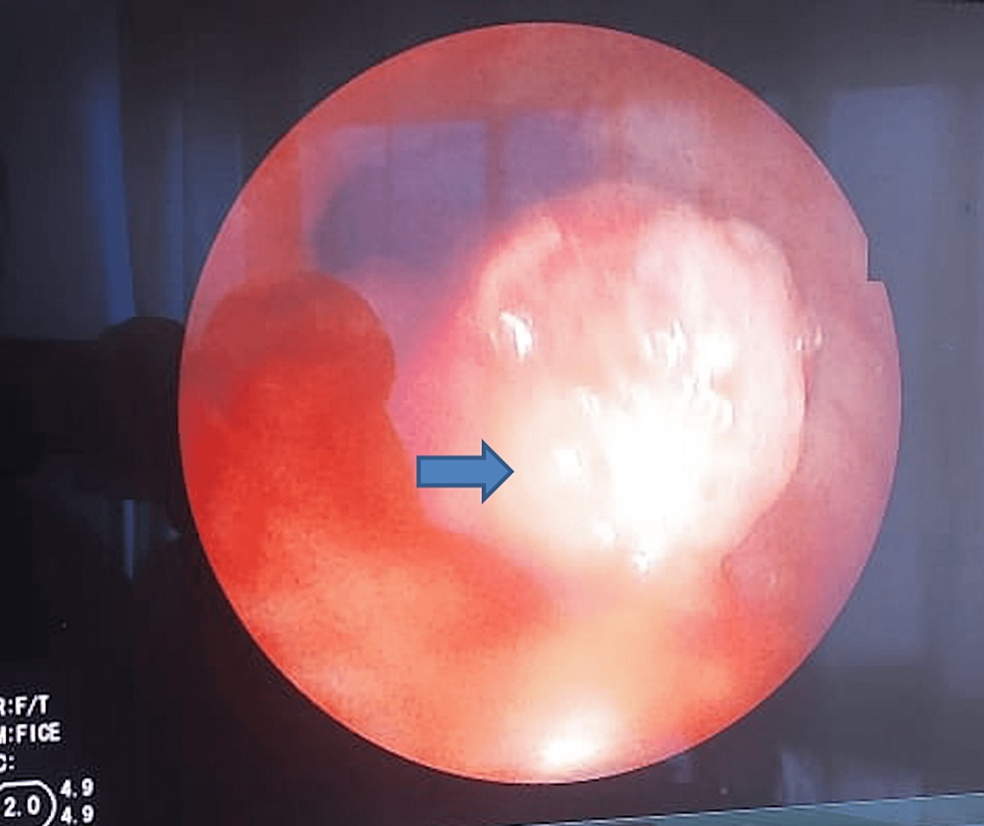
Bronchoscopy revealed an obstruction of the upper third occupying 45% of the tracheal lumen (blue arrow), bleeding easily on contact.

Bronchoscopic endoscopy (Figure 2) revealed an obstruction of the upper third occupying 45% of the tracheal lumen, bleeding easily on contact. Biopsies were performed at this level.

**Figure 3:**
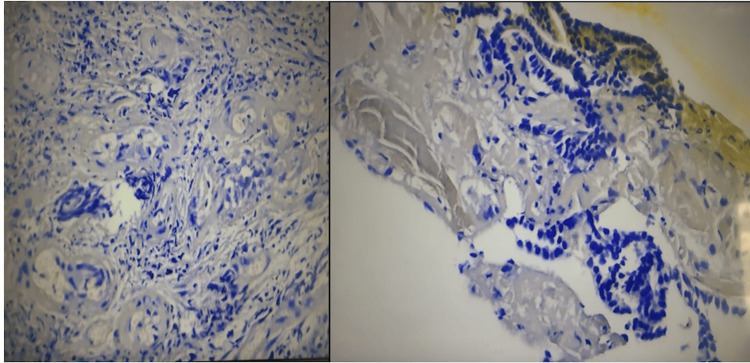
Histological aspect of a fleshy bud on a fragment associated with suspicious glandular formations

**Figure 4:**
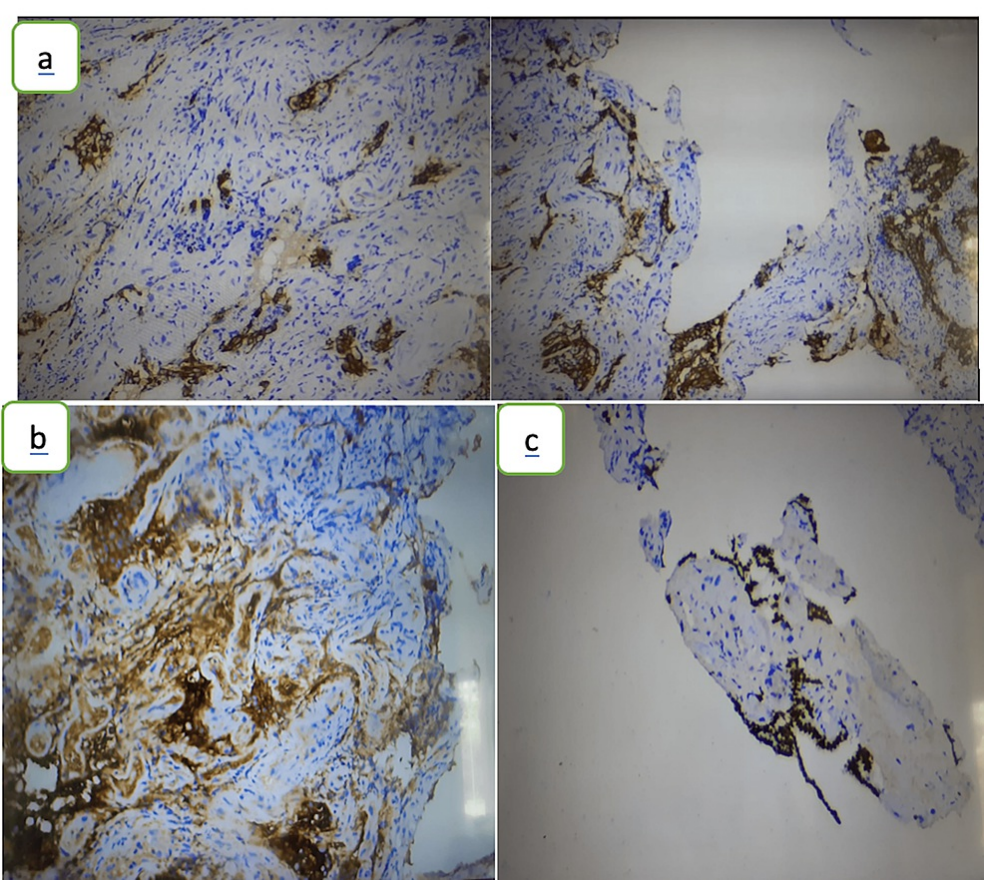
An immunohistochemical study: Positive marking of tumor cells by anti-CK7 antibody (a), anti-TTF1 antibody (b) and anti-thyroglobulin antibody (c).

The histological examination (Figure 3) revealed a histological aspect of a fleshy bud on a fragment associated with suspicious glandular formations. An immunohistochemical study (Figure 4) was carried out showing positive marking of tumor cells by anti-CK7 antibodies, anti-TTF1 antibodies, anti-thyroglobulin antibodies, and negative marking of tumor cells by anti-CK20 antibodies.

The presence of thyroid tissue confirmed the endotracheal localization of a papillary thyroid carcinoma.

This case was discussed in a multidisciplinary consultation meeting of oncology. Given his age, general state, ischemic heart disease, and cerebral localization, the patient was referred for palliative treatment.

## Discussion

Tracheal invasion occurs in a third of cases of locally invasive thyroid cancer, making it the third-most common site of local invasion after the recurrent laryngeal nerve; the site most frequently invaded is the infrahyoid muscles, and other common sites of invasion are the oesophagus and the larynx [2,3]. Laryngeal involvement is relatively rare and affects 12% of patients with locally invasive thyroid cancer [3]. In the literature, a female predominance is noted [3,4], but some find a male predominance [5] or equal occurrence across genders [6]. When interviewing a patient with a goiter, the presence of pharyngolaryngeal functional signs, dysphonia, dyspnea, dysphagia, or cervical pain should raise suspicions of laryngo-tracheal extension. Hemoptysis can also be revealing as in our patient. These signs may be due to extrinsic or nerve tumor compression (laryngeal nerves), or intra-luminal pharyngolaryngeal or tracheal tumor extension. That's why fiber-optic laryngoscopy should be performed at the time of diagnosis and for the monitoring of these malignant thyroid tumors to look for a disorder of laryngeal mobility or a change in pharyngo-laryngeal reliefs. When this involvement is clinically suspected or found on a CT scan, a panendoscopy should be performed to assess intra-luminal extension (larynx, trachea, and esophagus) and thus make the right indication for surgical resection [3,5,6]. Biopsy of the tumor bud allows us to determine the pathological nature of the lesion and eliminate a cartilaginous tumor or a squamous cell carcinoma of the upper aerodigestive. In our patient, the diagnosis was made on bronchial fibroscopy. Several stages have been described in relation to the depth of tracheal invasion. Stage I corresponds to an invasion of the perichondrium, respecting the cartilages, stage II to a cartilaginous invasion or inter-annular ligaments, stage III to a submucosal extension, and stage IV to a mucosal or intraluminal extension [2]. Our patient was classified as stage IV given the endoluminal invasion. The treatment of differentiated carcinomas of the thyroid extended to the laryngo-tracheal axis combines surgery, radioactive iodine 131, lifelong replacement hormone therapy, and possibly external radiotherapy. The attitude adopted towards laryngo-tracheal extension is controversial. Some authors propose a macroscopically incomplete tumor resection sparing the laryngo-tracheal axis, completed by iodine 131 and/or external radiotherapy. Other authors report performing a chondro-mucous resection in a healthy area with or without reconstruction [7,8]. The prognosis of differentiated cancers of the thyroid with an invasion of the laryngo-tracheal axis is more favorable when the diagnosis is made early and the surgical resection is complete [2].

## Conclusions

The papillary thyroid carcinoma of the thyroid is generally characterized by slow growth and excellent long-term survival, however, it can present clinical challenges when it manifests in unusual ways. In some cases, the atypical manifestations of papillary carcinoma may lead to delayed or misdiagnosed cases, potentially affecting the treatment outcomes and patient prognosis. Tracheal invasion occurs in a third of cases of locally invasive thyroid cancer. The treatment of differentiated carcinomas of the thyroid extended to the laryngo-tracheal axis combines surgery and radioactive iodine 131. The importance of a thorough understanding of this disease and its various presentations is crucial to ensure optimal patient management and prognosis improvement.
